# A146 BENIGN GASTRIC ULCER COMPLICATED BY GASTROJEJUNAL FISTULA FORMATION HEALED WITH PROTON-PUMP INHIBITOR

**DOI:** 10.1093/jcag/gwac036.146

**Published:** 2023-03-07

**Authors:** R Winter, A Ilnyckyj

**Affiliations:** Department of Medicine - Gastroenterology, University of Manitoba, Winnipeg, Canada

## Abstract

**Background:**

We report a case of a benign gastric ulcer (GU) complicated by gastrojenunal (GJ) fistulization which healed with proton-pump inhibitor (PPI) therapy. It presented in an elderly malnourished patient.

**Purpose:**

To highlight this rare complication of benign peptic ulcer disease, as only 13 similar cases are reported. Additionally, our case is one of the few demonstrating medical treatment can effectively heal both the ulcer and GJ fistula, thus obviating the need for surgery.

**Method:**

A detailed chart review was conducted to summarize all salient clinical data. Using PubMed database, a comprehensive literature review identified similar cases using MeSH terms such as “gastrojejunal”, “fistula”, and “ulcer” under the case report filter.

**Result(s):**

An 83-year-old previously healthy woman presented with four weeks of weakness, abdominal pain, and nausea. She denied any overt GI bleeding or NSAID use. Physical exam demonstrated cachexia (36.6kg) with benign abdominal and rectal examinations. Initial workup revealed anemia (hemoglobin of 38g/L), iron deficiency, hypoalbuminemia (20g/L) and depleted B12 (108pmol/L). She was started on IV pantoprazole and transfused 3 units of red cells.

A gastroscopy revealed a giant, penetrating gastric ulcer at the lesser curvature. The ulcer base showed protruding tissue consistent with a segment of intestine (Figure 1 - left image). Examination to the distal duodenum was normal. Biopsies of the ulcer edge were negative for neoplasia. Urease testing of antrum and body samples were negative. CT scan demonstrated a 3.1 cm gastric ulcer with a linear tract communicating with the proximal jejunum, confirming the endoscopic impression.

Surgical consultation was obtained; conservative treatment was advised given her age and cachexia. Calorie intake was optimized and the patient commenced twice daily PPI. She rapidly gained 7 kg in the community, accompanied by normalization of albumin (33g/L) and B12 (293 pmol/L) levels. Hemoglobin remained stable near 100g/L after initial transfusion. At month three, repeat gastroscopy was performed. The ulcer and fistula had completely healed. (Figure 1 - right image).

**Image:**

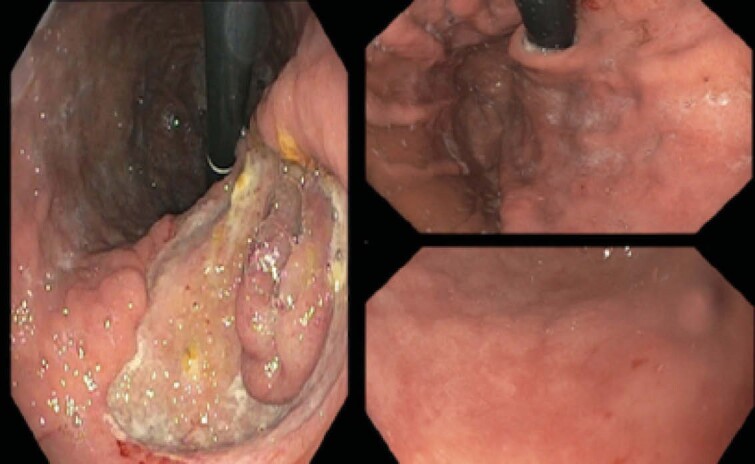

**Conclusion(s):**

Gastro-enteric fistulas complicating benign ulcers are rare. When described, other sites, such as gastroduodenal, are more common. Direct gastrojejunal fistulization is less favorable due to the anatomy of the mesentery. The literature reports only 13 gastrojejunal fistulas complicating benign ulcers. In other cases, unique risk factors are reported (malignancy, prior gastric surgery). Only 3 of 13 reported cases resolved with medication alone. The overwhelming majority required surgery. Notably, many of the case reports pre-date the introduction of proton-pump inhibitors.

The etiology of our patient’s ulcer was benign but remains undefined. ASA use was excluded on history, and H. pylori sampling was negative. We speculate profound malnutrition placed her at risk for poor healing and thus contributed to the extensive disease complicated by fistula.

**Please acknowledge all funding agencies by checking the applicable boxes below:**

None

**Disclosure of Interest:**

None Declared

